# Tired of climate targets? Shift focus of IPCC scenarios from emission and growth targets to policies

**DOI:** 10.1111/nyas.14900

**Published:** 2022-09-08

**Authors:** Ivan Savin, Jeroen van den Bergh

**Affiliations:** ^1^ Institute of Environmental Science and Technology Universitat Autònoma de Barcelona Barcelona Spain; ^2^ Graduate School of Economics and Management Ural Federal University Yekaterinburg Russian Federation; ^3^ ICREA Barcelona Spain; ^4^ School of Business and Economics & Institute for Environmental Studies VU University Amsterdam Amsterdam the Netherlands

**Keywords:** climate policy, degrowth, economic growth, IPCC, post‐growth

## Abstract

Climate change has revived the debate on growth‐versus‐environment. In line with this, recently it has been proposed to shift the target focus of IPCC scenarios from emissions to post‐growth. We argue here that this confounds ends and means, since while reduction of growth may be an outcome of good climate policies, it should not be a goal in itself. In fact, a post‐ or degrowth goal would mean an ineffective and costly way to reduce emissions. Instead, we suggest that the debate about pursuing economic growth versus achieving climate goals will become more transparent and policy‐relevant through refocusing scenarios from targets to policy.

## Graphical abstract

Recently it has been proposed to shift the target focus of IPCC scenarios from emissions to post‐growth. We argue here that this confounds ends and means, since while reduction of growth may be an outcome of good climate policies, it should not be a goal in itself. Instead, we suggest that the debate about pursuing economic growth versus achieving climate goals will become more transparent and policy‐relevant through refocusing scenarios from targets to policy.

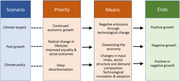

## INTRODUCTION

Since it is widely feared that stringent policies for climate change mitigation will affect economic growth, the goal of growth itself poses a serious challenge for getting critical social and political support to implement these policies. Given the uncertainties about the relationship between climate policy and economic growth, it is hardly surprising that one can find a wide range of opinions about it, ranging from unconditional progrowth, through green growth and agrowth, to unapologetic antigrowth or even anti‐capitalism.^1^ This is part of a broader and longstanding debate on growth‐versus‐environment,^2^ which only recently has zoomed in on growth‐versus‐climate (policy). Here, we examine the relevance of the different positions for scenario development and analysis aimed at informing climate policy making.

In a recent opinion article, Hickel et al.^3^ criticize “scenarios reviewed by the Intergovernmental Panel on Climate Change (IPCC)” for making unrealistic assumptions about the potential to scale up bioenergy with carbon capture and storage technologies. They propose to complement this approach with so‐called “post‐growth” targets, where post‐growth is really a euphemism for degrowth. While we understand that for many participants in the debate on climate solutions it appears attractive to translate the friction between economic growth and emissions reduction into an explicit limits‐to‐growth or degrowth target, we will argue that this confuses means with ends. Instead of focusing on specific emission, technological or growth targets, there is an urgent need to study scenarios that describe concrete policies aimed at achieving the emission targets. Only this will allow assessment of whether and how policy‐induced changes in choices by consumers, producers, investors, and innovators can decouple growth and emissions or, alternatively, translate into reductions in, or even negative, economic growth.

We agree with Hickel et al. that scenarios need to explore a wide set of policy options, and that relying heavily on negative emissions, as many positive growth scenarios do, is risky. To date, the majority of IPCC scenarios focus on end‐of‐century targets, which allows cumulative emissions to overshoot the CO_2_ budget prior to 2100, in turn increasing the risk of dangerous climate change, resulting in huge costs for society.^4^ A recent study examines limiting this overshoot, which translates into lower cumulative mitigation costs.^5^ We also recognize that, and understand why, Hickel et al.’s post‐growth focus rings a bell with many people in society. Hence, it merits serious attention in scientific analysis. One might oppose, perhaps, that degrowth currently has little support in actual politics. Nevertheless, its presence in intellectual debate on climate solutions is steadily increasing—for good or bad.

However, we feel that by assuming the main role for post‐growth, that is, through setting an ex‐ante degrowth target, Hickel et al. adopt an overly pessimistic and dogmatic approach. Indeed, their approach assumes beforehand that decoupling of emissions and economic growth will be impossible, even with (not yet implemented) stringent policies. Instead, it makes more sense to include scenarios not requiring permanent growth (and neither permanent degrowth) next to scenarios with growth, as these are more likely to come true given current economic and political systems—and there is no sign yet that this is soon going to change.

Political unrealism aside, Hickel et al.’s proposal also lacks a solid scientific basis due to confusing means with ends. Indeed, policies aimed at reducing emissions may result in negative growth—one means among many to realize emissions reduction, next to technology, input mix, supply structure, and demand composition—but negative growth is not, or at least should not be, an end in itself. More importantly, negative growth does not provide any guarantees for a low‐carbon economy and may result to be an ineffective and costly way (e.g., sacrificing considerable welfare) of reducing emissions. Moreover, by proposing to replace climate targets with post‐growth targets, the authors fall into the trap of continuing the target approach without offering a solid framework for balancing all types of means to achieve the emission targets. Instead, we should be more precise about the distinction between means and ends in the context of economic growth and climate, which requires shifting the scenario focus from targets to policies.

Incidentally, the idea of “IPCC scenarios” needs nuancing. The climate‐change research community generates scenarios that are then reviewed and discussed by the IPCC. Moreover, the research community produces not only scenarios with negative emissions but also policy scenarios.^6^, ^7^ Against this background, we focus our attention on the tendency of the IPCC to give relatively greater attention to scenarios focused on targets,^8^, ^9^ while scenario studies explicitly addressing policies are more scarce and have appeared only recently.^10^, ^11^, ^12^ This may largely be because the IPCC primarily relies^13^ on integrated assessment models (IAMs). Many so‐called policy scenarios in these models are implemented using carbon budget constraints or Paris Agreement NDCs, which assume that policies are implemented consistently with these but without specifying any policies in the models.^14^, ^15^ Moreover, IPCC scenarios include growth pathways as assumptions, reflecting an unwillingness to sacrifice (some) growth for the sake of emission reduction. This runs the risk of arriving at overly optimistic conclusions, as the model's assumptions very much drive its outcomes.^16^, ^17^ Surprisingly, this has not received much attention in criticisms of IAMs.^18^, ^19^

In the remainder of this article, we critically examine the use of both emission and growth targets in climate policy studies. While we understand the attraction of setting such targets, we will argue instead that it is better to focus on *policy scenarios*, for at least two reasons. It allows depiction of the full cycle from policies to economic impacts, including growth and, finally, emissions. And it will provide realistic perspectives and advice to policymakers about what to expect from policies in terms of offering definite climate solutions.

## CONFUSION BETWEEN MEANS AND ENDS

In our view, stressing economic decline or “downsizing the economy” as an inevitable outcome of climate solutions is neither scientifically warranted nor politically wise. Hickel et al. propose that high‐income countries should just stop growing and concentrate on redistribution of wealth and improving social outcomes. They suggest that “post‐growth scholarship demonstrates that by organizing the economy around principles of equity and sufficiency, societies can deliver high levels of human well‐being with significantly less energy and resources than rich countries presently use.” But the truth is that there is no experience with post‐growth policies in the real world, while only very few studies have examined them in a hypothetical model context.^20^, ^21^, ^22^ These studies typically focus on defining a normative level of consumption to achieve emission targets, not on which climate policies can bring about the required reduction in consumption, and hence not on the political feasibility of such policies. In fact, Millward‐Hopkins et al. state that “[we] entirely avoided the most difficult question: how we could get from the current global situation of vast inequalities, excess and inefficient energy‐use to one where decent living standards are provided universally and efficiently.”

**FIGURE 1:**
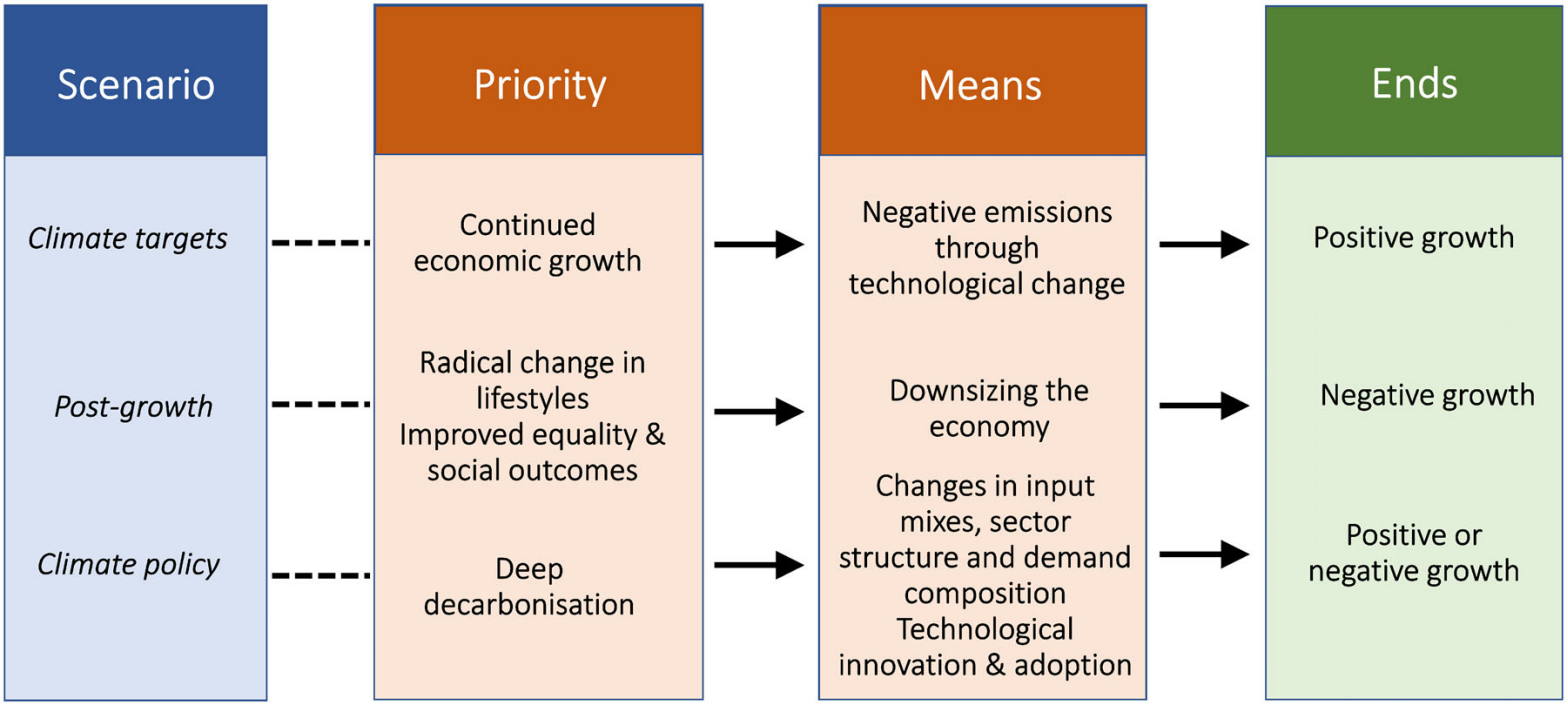
Shifting the climate scenario focus from ends to means.

While scenarios assuming continued growth in IPCC reports could be interpreted as a willingness to take a risk with the climate by over‐relying on the technological progress, degrowth scenarios are taking a similar, if not larger, risk by assuming unprecedented radical social change without offering a clear strategy for, or policy on, how to accomplish it. In fact, both the emission‐target scenario and the post‐growth scenario can be judged as insufficiently reflecting the means–ends dichotomy—as visualized by the top rows in Figure 1. As a result, it is difficult to test their effectiveness or even political realism. Among others, the post‐growth scenarios require greater public spending and record deficit‐to‐GDP ratios, making them very costly and, thus, unattractive to politicians and voters.^4^ On top of this, these scenarios seem to be disconnected from the literature on climate policy design and support, and thus lack a coherent view on which particular policy instruments can most effectively bring about changes toward a low‐carbon economy.^23^ One explanation for this may be that modeling climate policy with cause–effect chains, including policy instruments, economy, and emissions, is difficult; it is, therefore, easier to treat emission trajectories as the result of broad technological or lifestyle strategies, regardless of which policies can achieve these.^24^ But such easy model solutions are not what policymakers need. As for emission‐target scenarios, they often involve breaking down aggregate, national targets rather arbitrarily into a set of targets for production sectors (or even demand).^25^ This overlooks heterogeneity of abatement options and costs between sectors, which obstructs systemic policy solutions that aim to guarantee effectiveness through limiting intersectoral shifts and rebound, as well as achieving a smooth transition through a selection of the most efficient emission‐reduction options.^26^

The debate about growth versus environment—recently zooming in on growth versus climate—lingers on exactly because neither side can provide definite evidence for their view. Indeed, it remains uncertain whether decoupling of GDP and CO_2_ emissions is possible. It may seem unlikely if one looks at the past but given that the stringency of the implemented policies so far has been weak, we have actually little to no evidence about the impact of serious policies. Recent studies indicate that some OECD countries show evidence of decoupling.^27^, ^28^ Still, one cannot conclude that climate policy, notably in the form of stringent systemic regulation and pricing, will be unable to enforce decoupling. The question is also why one would be less optimistic about decoupling than about the possibility to convince people to radically change their lifestyle and associated consumption?

Why not avoid either uncertain scenario and instead opt for a third policy approach that does not assume any ex‐ante growth or degrowth? This could involve policies that seriously regulate all consumers and producers to reduce their emissions. This will give rise to many changes other than (possibly) adapting the level of consumption and production, the emphasis in post‐growth scenarios. A policy focus can be judged as more scientific given that it is undogmatic about growth and consistent with the means–ends chain from policy through behavioral changes in all activities to emissions reduction. The latter is visualized by the bottom row in Figure 1. The policy approach puts deep decarbonization as the priority, not economic growth or degrowth. It, therefore, allows identifying the combination of solutions that reduce emissions effectively, efficiently, and equitably, and hence guide a smooth transition. In addition, this policy approach has the advantage of allowing to test whether the associated concrete policies can count on sufficient public support, or how such support can through clever adaptation of policy design gradually be improved.^29^

## POST‐GROWTH LACKS ATTENTION FOR POLICY SUPPORT

We have the impression that policy support is not the main concern in post‐growth thinking, which then runs the risk of depicting a fantasy world. This is in line with post‐growth views occupying a marginal position in the political spectrum, even among left‐wing parties.^30^ To increase the feasibility of policy implementation, one best avoids stressing the negative growth implications of policies as these are uncertain and unlikely to generate enthusiasm among the broader public. Take, as an example, the COVID‐19 lockdown measures, which can be considered a “natural experiment” of degrowth that sparked resistance in many countries.^31^, ^32^ Instead, we should try to ensure society is less concerned about economic growth, which makes a lot of sense in view of the uncertainties about decoupling under stringent policies and the widely accepted shortcomings of GDP as a social welfare indicator.^33^ Indeed, if the indicator is not relevant, one should be indifferent about its trend or fluctuation over time. This growth indifference would prepare people better to accept policies for a transition to a zero‐carbon future, compared to confronting them with an explicit negative‐growth target. If climate policies result in reduced growth, so be it. But presenting negative growth *as an inevitable outcome upfront* is unscientific as well as politically unwise.^34^ The prospect of negative growth—that is, downsizing the economy—risks that many voters and politicians withdraw support for stringent climate policy.

One should also recognize that any degrowth targets are likely ineffective as they do not automatically select the most emission‐intensive activities to be downscaled. They could well make the economy less efficient overall. In addition, consumption sufficiency measures can count on a considerable rebound, thus reducing the net effectiveness of the post‐growth strategy.^35^ Moreover, degrowth in rich nations might—in view of the interconnectedness of the global economy through trade and investments—cause a serious decline in the economies of developing countries,^36^ which could increase global inequity. This would evidently reduce political support for it. Altogether, the normative degrowth proposal may sound sympathetic to many, but is rather simplistic, lacking a solid theoretical and empirical basis.

## POLICY SCENARIOS AND SYSTEMIC EFFECTS

Post‐growth studies of climate solutions often involve arbitrary assumptions, such as *x*% reduction in energy consumption^37^ or *y*% reduction of working time.^38^ Such targets sound more like wishful thinking rather than evidence‐based science. Instead of simply assuming certain changes in behavior, we need to link them to policy instruments that can stimulate the relevant systemic changes. Incidentally, in the recent literature, several studies have appeared which model lifestyle changes.^39^, ^40^, ^41^ Such changes are, however, typically modeled as exogenous and not as the outcome of policies—and hence their realism or political feasibility remains unclear.^42^

What post‐growth thinking seems to overlook is that systemic climate policies have multiple effects: input substitution, innovation and adoption of low‐carbon technologies, and changes in sectoral, trade, and demand structure. Moreover, they encourage reductions in both consumption and production, that is, automatically slowing down economic growth. This is particularly true if we put a hard emission limit on the economy, such as through an emissions trading system.

In view of this, we recommend that IPCC devotes more attention to effective policies from a systemic perspective, through accounting for positive and negative synergies of instruments in a policy mix, and by assessing overall effectiveness inclusive of energy/carbon rebound, carbon leakage, and green paradox.^43^

Earlier theoretical and empirical evidence regarding the effectiveness of combining instruments—comprising performance and technical standards, carbon pricing, adoption subsidies, innovation support, and information provision—was reviewed by van den Bergh et al.^44^ Negative synergies were identified between technical standards or targets combined with a carbon market. This is because targets distort permit prices, leading to a *waterbed effect* (i.e., emissions, instead of being reduced, shift to other sectors). Combining carbon pricing with innovation subsidies, in contrast, results in positive synergies, since—thanks to such subsidies—promising but still expensive technologies can survive the pressure from carbon pricing, which helps to avoid early technology lock‐in. Information provision can also create positive synergy with carbon pricing, for example, by making consumers more aware of the emissions associated with consumption, by informing them about instrument performance, or by stimulating social imitation of low‐carbon options.^45^ Taking into account that a simpler policy mix has a higher potential or international harmonization, van den Bergh et al. recommend keeping climate policy transparent by limiting the complexity of the policy mix, namely through combining a carbon market with innovation support and information provision.

Incidentally, toward the end of their article, Hickel et al. suggest a set of policy interventions, which they frame as a “post‐growth alternative.” Many of their examples, such as public transport or energy‐efficient buildings, are already part of the mainstream discussion.^46^ In addition, many of their suggestions, and possibly their clearest degrowth examples—minimizing food waste, reducing industrial agriculture, creating 15‐min compact urban centers, and decreasing average dwelling size, take the form of targets, not policies. This well illustrates the confusion between policies and targets, that is, means and ends. In addition, it is not evident that these targets will make a significant contribution to total emissions reduction.

Surprisingly, the proposed list of actions omits explicit mention of carbon pricing, the only instrument able to consistently control direct and indirect emissions of decisions by producers and consumers as well as long‐term innovation effects.^47^ This omission is particularly unfortunate, as carbon pricing is well‐equipped to limit rebound effects that are a likely outcome of many degrowth targets and measures. Some recent reviews and empirical studies for the United States and Europe show that such a rebound can reach up to 100%, underpinning the criticality of pricing policies.^48^, ^49^ Moreover, carbon pricing arguably offers the best potential for international harmonization of climate policy, essential for realizing much more stringent policies in all countries.^50^, ^51^ In fact, the EU's carbon pricing, in the form of its emission trading system, is currently the main climate policy that has been harmonized among 31 countries. These two strengths—limiting rebound and policy harmonization—have been recently found among the main reasons why scientists from a wide range of disciplines (not only economics but also engineers, legal scholars, psychologists, and industrial ecologists) support this policy instrument.^52^ In addition, it provides funds (in the form of a carbon tax or permit revenues) to distribute among low‐income or otherwise vulnerable groups to both compensate regressive effects^53^ and make the policy politically more feasible.^54^ Hickel et al. instead focus very much on weaker and nonsystemic instruments, which in our view at best serve a supplementary, rather than core, role in quick and substantial emissions reduction.

Our hope is that a shift in attention in IPCC reports to policy scenarios would facilitate a similar shift in focus of the post‐Paris negotiations. Whereas COP26 has produced new targets—such as for methane, deforestation, and coal—these are not guaranteed to be matched by effective policies. This was an immediate criticism by NGOs and other stakeholders, who correctly pointed out that even past targets and pledges are still awaiting consistent national policies. Shifting future COPs to negotiate agreements about policy rather than about targets will foster policy consistency and harmonization among nations. This stimulates policymakers to act now instead of endlessly discussing future targets.^55^ In turn, this will facilitate a transition to the next—and hopefully final—stage with globally consistent and stringent policies that bring emissions swiftly down to zero, regardless of their implications for growth.

## AUTHOR CONTRIBUTIONS

The authors contributed equally to this paper.

## COMPETING INTERESTS

The authors declare no competing interests.

### PEER REVIEW

The peer review history for this article is available at: https://publons.com/publon/10.1111/nyas.14900.
